# Diabetic Foot Infections: The Diagnostic Challenges

**DOI:** 10.3390/jcm9061779

**Published:** 2020-06-08

**Authors:** Chiara Lauri, Antonio Leone, Marco Cavallini, Alberto Signore, Laura Giurato, Luigi Uccioli

**Affiliations:** 1 Nuclear Medicine Unit, Department of Medical-Surgical Sciences and of Translational Medicine, “Sapienza” University of Rome, 00161 Rome, Italy; alberto.signore@uniroma1.it; 2 Department of Nuclear Medicine and Molecular Imaging, University of Groningen, University Medical Center Groningen, 9700 Groningen, The Netherlands; 3 Department of Radiological and Haematological Sciences, Fondazione Policlinico Universitario A. Gemelli, ISCSS—Università Cattolica del Sacro Cuore, 00168 Rome, Italy; antonio.leonemd@gmail.com; 4 Wound Care Department of Medical-Surgical Sciences and of Translational Medicine, “Sapienza” University of Rome, 00161 Rome, Italy; marco.cavallini@uniroma1.it; 5 Diabetic Foot Unit, Department of Systems Medicine, University of Rome Tor Vergata, 00133 Rome, Italy; lauragiurato@yahoo.it (L.G.); luccioli@yahoo.com (L.U.)

**Keywords:** infection, diabetic foot, imaging, WBC scintigraphy, [^18^F]FDG PET/CT, MRI

## Abstract

Diabetic foot infections (DFIs) are severe complications of long-standing diabetes, and they represent a diagnostic challenge, since the differentiation between osteomyelitis (OM), soft tissue infection (STI), and Charcot’s osteoarthropathy is very difficult to achieve. Nevertheless, such differential diagnosis is mandatory in order to plan the most appropriate treatment for the patient. The isolation of the pathogen from bone or soft tissues is still the gold standard for diagnosis; however, it would be desirable to have a non-invasive test that is able to detect, localize, and evaluate the extent of the infection with high accuracy. A multidisciplinary approach is the key for the correct management of diabetic patients dealing with infective complications, but at the moment, no definite diagnostic flow charts still exist. This review aims at providing an overview on multimodality imaging for the diagnosis of DFI and to address evidence-based answers to the clinicians when they appeal to radiologists or nuclear medicine (NM) physicians for studying their patients.

## 1. Introduction

Diabetic foot infection (DFI) is a common complication of longstanding diabetes, and it is associated with considerable morbidity, the increased risk of lower extremity amputation, and a high mortality rate [1]. The development of DFI derives from a complex interplay among peripheral neuropathy, peripheral arterial disease (PAD), and the immune system.

Neuropathy is the most prominent risk factor for diabetic foot ulcerations (DFU). Motor neurons damage results in foot deformities that contribute to the injury of foot tissues and bones. Sensory neurons damage leads to a loss of protective sensation. Therefore, neuropathic patients could develop skin ulcers that might remain unrecognized for a long time, thus exposing the adjacent soft tissues to the colonization of pathogens and causing a soft tissue infection (STI). If not promptly identified and treated, the infection could spread to the underlying bone and cause osteomyelitis (OM).

PAD further facilitates micro-organisms invasion and rapid progression to infection, since insufficient tissues oxygenation might impair the healing of ulcers, creating an optimal substratum for the colonization of pathogen. In addition to this, PAD reduces granulocytes migration and antibiotic penetration into infected site, thus contributing to the spread of infection and complicating its therapeutic management. Moreover, patients with severe PAD are prone to sudden ischemia resulting from arterial thrombosis with consequent critical limb ischemia and an increased risk of amputation [2,3]. Indeed, both ischemia and infection are the most important factors in determining the prognosis of foot ulcerations, since patients with PAD and infection show more severe comorbidities and worst clinical outcomes compared with the classic “neuropathic foot patients” [4]. Uncontrolled hyperglycaemia represents another pivotal aspect in the pathogenesis of DFI being responsible of an impairment of both cell-mediated and humoral immune response mainly characterized by altered leukocyte’s functions, reduced chemotaxis, and phagocytosis proprieties [5,6].

A prompt identification of foot ulcers, STI, and OM and an accurate evaluation of the extent of the infective process is crucial for prognostication of the patients and for planning the most appropriate treatment that usually requires a combination of metabolic control, medical treatment with specific antibiotic regimen, and surgical approach. The International Working Group (IWGDF) and the Infectious Diseases Society (IDSA) proposed a single scheme to assess the presence and the severity of infection [7,8], and this classification is currently applied for predicting the need for hospitalization, the likelihood of undergoing lower extremity amputation, and other adverse outcomes [9]. However, in the latest update of these guidelines, OM and STI have been addressed separately, since they are two distintict conditions, although they may coexist in the same patient, with different diagnostic, therapeutic, and prognostic implications [10].

Clinical suspicion through a comprehensive history and physical exam are the starting points for the diagnosis of DFI, which is validated by a complete laboratory evaluation, microbiologic assessment, and imaging.

Clinical diagnosis of superficial STI is based on the presence of at least two local signs of inflammation: rubor, calor, dolor, tumour, or purulent secretion. Other secondary features suggestive of infection may be present, such as friable or discolored granulation tissue, necrosis, and failure of the wound to heal [11]. Clinical manifestations of acute deep infection include abscess, necrotizing fasciitis, and gangrene. In those cases, the infection process may involve one or more foot compartments and may require a first urgent surgical treatment and eventually distal revascularization to reduce the amputation level [12].

The development of an OM is one of the most serious and disabling complications of diabetes, being associated with prolonged antibiotic therapy and hospitalization, as well as higher re-infection rates and risk of amputations compared with patients with STI, resulting in high social costs [13].

Diagnosing OM is sometimes a challenge for the clinicians, since it may occur in the absence of local or systemic signs of infection and inflammation, especially in chronic infections. Several wound characteristics, in particular the width and depth of the lesion, may be helpful in predicting the presence of bone infection. A lesion’s surface greater than 2 cm^2^ has a sensitivity of 56% and a specificity of 92% for the diagnosis of OM. Similarly a deep ulcer over 3 mm is significantly associated with an underlying OM in comparison with a more superficial one (82% versus 33%) [14].

Another diagnostic criterion is represented by the possibility to reach the bone with a blunt at the base of the lesion, the “probe-to-bone test”. Combining the results of the probe-to-bone test with those of plain radiography improves the overall diagnostic accuracy of OM [15,16].

However, the gold standard for the definitive diagnosis of OM still remains the bone biopsy that provides histological and microbiological information and, at the same time, it is useful to determine the susceptibility to various antibiotics [7]. Although bone biopsy is the more accurate technique in identifying the pathogenic germs, it is an invasive procedure, and it is not always feasible. However, a culture of deep soft tissue that is in direct contact with the bone shows a good correlation with bone biopsy in identifying the responsible pathogen and, therefore, this approach may be useful in alternative to bone biopsy [17].

Imaging offers the possibility to diagnose DFI by using a less invasive approach that is complementary to physical examination, laboratory, and microbiological evaluations. A wide panel of modalities may be very helpful for the clinicians to better understand whether the patient has a STI, OM, or sterile inflammation that is a hallmark of Charcot osteoarthropathy, for example (Table 1). To achieve an accurate differential diagnosis is mandatory in the optic of promptly starting an appropriate treatment reducing the need for hospitalization and the risk of major amputations, but univocal consensus on diagnostic criteria for imaging modalities still does not exist.

This review aims at providing an overview of radiologic and nuclear medicine (NM) modalities able to achieve an accurate differential diagnosis between the different kinds of DFI and to guide therapeutic strategies.

## 2. Surgical Management of DFI: How Can Imaging Be Useful?

Surgical management for diabetic foot (DF) deformities and complications is a critical aspect in dealing with these patients. Understanding of the DF ‘syndrome’ has improved the approach to diabetic patients affected by a complicated foot. In the last decades, we observed an increasing interest in developing less invasive surgical procedures as alternatives to major lower extremity amputation. They are focused on local resections and the drainage of infected underlying soft tissue, toes, and metatarsal heads for neuropathic or neuroischemic complicated DF [18,19]. In this optic, imaging plays a crucial role in diagnosing the infection and defining its extent, aiming at selecting those cases candidate to more conservative approaches.

Structural deformities and high plantar pressures are a predisposing risk factor to diabetic foot ulceration (DFU) [20,21,22,23]. Common deformities include hammertoes, prominent metatarsal heads, hallux limitus, Charcot foot, and previous toe or partial foot amputations [24]. Each leads to high pressures that contribute, in the case of an insensitive DF, to tissue inflammation and ulceration. Ameliorating these high pressures by structurally realigning or removing bony prominences is the rationale for foot surgery. In the presence of infection, phlegmon, and/or OM, surgery becomes a critical urgent component of care [25]. A proposed scheme for classifying the types of foot surgery in diabetic patients refers to the presence of open wounds and their acuity [26]:

- Prophylactic procedures are those performed in neuropathic patients to reduce the risk of ulceration or recurrent ulceration in the absence of open wounds;
- Curative surgery when cutaneous ulcers are present is often performed to provide a cure by joint resection, removing underlying bony prominences (surgical decompression), osteomyelitis, or by draining underlying abscesses or phlegmons;
- Urgent procedures are performed for severe deep or ascending infections (infectious gangrene, necrotizing fasciitis, etc.) to control the progression of infection. These procedures are performed emergently and usually consist in wide open drainages or minor amputations at the foot level.

In daily clinical practice, curative and urgent procedures are most frequent, since usually patients arrive to a surgical referral with an active more or less complicated DFU.

When dealing with deep infected cutaneous ulcers, the primary principle in treating surgical infection is source control. Most infected DFUs respond well to local debridement, the administration of culture-specific antibiotics, and offloading of the foot with specific footwear. Some develop a rapid spread of infection along the tissue planes and tendon sheaths and present with local tissue necrosis, spreading cellulitis and systemic inflammatory response [27].

According to the T.I.M.E. (Tissue, Infection, Moisture and Edges) procedure, source control includes the resection and/or debridement of any dead/infected tissue/bone and avoid fluid stasis by draining any hidden infected site [28]. However, Time stands also for “do not waste Time” in referral the patient to specialists who can better deal with the patient’s need and also stands for “Timing”, indicating untimely or adequate choice of procedure (for example, limb revascularization) to treat the patient at its presentation. Since deep foot infection can potentially be limb threatening without timely intervention, delay will lead to further tissue loss. In this case, we can state that “Time is Tissue”.

The endpoints of curative approach to deep foot ulcer and osteomyelitis are:

- Treat and cure the infection;
- Reduce pain (not always present because of neuropathy);
- Retain foot and allow best function (rehabilitation);
- Reduce recurrency.

Radical surgical resection, including healthy bone and soft tissue, is sometimes required and must follow an “oncologic approach” in the case of deep foot infections and OM [29,30], since they are difficult to treat and they could relapse.

Our understanding of the pathophysiology has been greatly improved by the biofilm model, which explains the wide variety of symptoms, courses, and the complex therapeutic management. The pathogens first form the surface layer of colonies, which then multiply into a three-dimensional structure. This biofilm structure offers the bacteria protection from mechanical influences and makes it harder for antibiotics, the body’s own defensive cells, and antibodies to penetrate, functioning as a diffusion barrier. The pathogens pass from a planktonic, free-floating phase with a high metabolic rate and rapid multiplication into a sessile form with greatly reduced metabolism and slowed biological reactions. This phenotypic change makes them more resistant to antibiotics compared to planktonic counterparts, since cellular growth within biofilms produces a matrix that protects the pathogens from the immune system and antimicrobial drugs. In OM and prosthesis-related infections, it has been calculated that this particular type of growth can reduce their sensitivity to antibiotics by a factor of 10^3^ [31]. The time required for a mature biofilm formation is about 24–48 h [32]. Mechanical forces of surgical debridement are effective in disrupting that matrix, exposing bacteria to the effects of antibiotics and body’s immune response. Therefore, with a surgical medication, we can realize a therapeutic window of 1–2 days where a sharp debridement should be repeated in order to remove all instable tissues and biofilm covering the wound bed.

**Figure 1:**
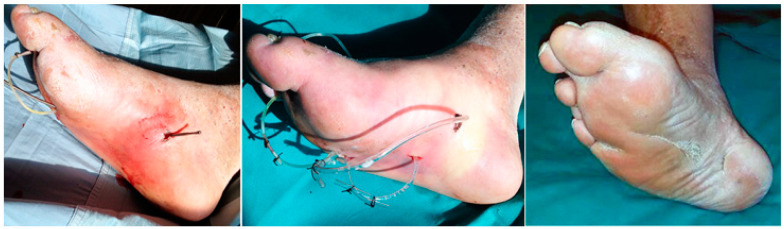
Extended plantar phlegmon. Left: With a probe, it is possible to follow the real spaces produced by the phlegmon spread along tissue plans. Where the end of the tract becomes superficial toward the skin, interposed tissues and the skin are pierced and incised in order to pass through the probe. Middle: A silastic tube is, thereafter, anchored to the probe in order to pass it backward along the fistula tract. Once this drainage is passed, the two ends are tied together with two silk stitches in order to construct the ulcer piercing ring (UP ring). Right: A diabetic foot ulceration (DFU) completely healed after 8 months in an out patient facility with daily medications and irrigations and with occasional antibiotic therapy and resulting with a small plantar scar. (Courtesy of Marco Cavallini).

All foreign bodies including screws and stitches must be removed, since they might be biofilm carriers. Any infected tendons and bone should be cleaned and irrigated in order to remove necrotic and/or infected tissues. The remaining tissues must be viable and well-perfused. There are no objective criteria for defining bone resection limits; therefore, it remains an individual decision of the surgeon, but generally, it should be up to when a hard bone is touched with the surgical instrument [33]. In some cases, non-infected bones must be removed or reduced in order to relieve the pressure to the underlying ulcerated cutaneous plane. The size of the defect produced by the procedure is not a primary consideration; only the vascular supply should be evaluated and preserved. What happens next depends on how radical the débridement and resection has been. Thereafter, the most important aspect is the management of dead space, which, if not treated properly, may lead to the early recurrence of infection and inadequate rehabilitation, especially if it involves the foot plantar surface. Surgical drainage is mandatory for the prevention of any fluid or exudate stasis that might be responsible for persistent bacterial contamination, biofilm, or infection and wound-healing impairment and delay [34] (Figure 1).

Concluding, the surgical management of DF complications is challenging and it requires an appropriate diagnosis in order to correctly identify the problem and to promptly start an adequate and a personalized treatment for the single patient. An interdisciplinary approach derived from close collaboration between clinicians, surgeons, radiologists, NM physicians, microbiologists, podiatrists, and nurses is mandatory.

## 3. Radiological Modalities for Imaging DFI

Although the reference standard for the diagnosis of diabetes-related OM still remains bone biopsy, the diagnosis is largely based on the presence of clinical and laboratory findings such as an erythrocyte sedimentation rate (ESR) >70 mm/h, and a positive result of a probe-to-bone test (palpation of bone in the depths of infected pedal ulcers) [10,11]. However, it should be kept in mind that (1) an ESR of more than 70 mm/h is highly specific for OM, but has a sensitivity of only 28% [35], and (2) the reliability of the probe-to-bone test may vary with the performing clinician’s experience and ulcer location [10,11,35]. In addition, the benefit of this test is substantially influenced by the pre-test probability of the patient having an OM or not. A positive probe-to-bone test suggests the diagnosis in a high-risk patient. A negative test indicates a low probability of OM in a low-risk patient [36,37]. Hence, the diagnosis of DFI may be difficult when based on clinical and laboratory findings alone. Advanced imaging of the foot has improved our ability to evaluate the possibility of OM, and it may be helpful for the diagnosis and definition of deep or soft-tissue purulent collections.

Radiography and magnetic resonance imaging (MRI) are the most commonly used radiological modalities to evaluate the DF infective complications. Ultrasounds can be employed for guiding the aspiration of suspect fluid collections or removing foreign bodies; however, it is not currently recommended by the IWGDF [10] and the diabetic foot guidelines of the American College of Radiology [38]. Computed tomography (CT), despite its higher sensitivity compared with radiography and MRI in detecting cortical erosions, periosteal reaction, small sequestra, soft tissue gas, and calcifications within sites of chronic osteomyelitis, plays a limited role in the imaging of diabetic patients with suspected OM or STI of the foot [38]. The main disadvantages of CT are the low soft tissue contrast resolution and the inability to detect the bone marrow edema seen in the early stages of infection. If MRI is contraindicated or unavailable, post-contrast CT may be used to detect soft-tissue and osseous abscess formation. However, the risk of use of iodinated contrast in diabetic patients should be taken into account, as diabetic nephropathy progressing to end-stage renal disease is commonly a comorbidity in patients with diabetes [39].

### 3.1. Radiography

The sensitivity of radiography is rather low in this setting, since radiographic findings of DF infective complications can be undetected for up to four weeks after the onset of infection, and these changes can be caused by Charcot osteoarthropathy and other disorders such as gout [40,41]. However, radiography should be the first-line imaging modality in any patient with suspected infection. It is cheap, widely available, and when radiographic findings such as demineralization, bone resorption, cortical destruction, periosteal reaction, bowing, or the obliteration of fat stripes and fascial planes, arthropathic changes, and the presence of soft tissue gas and foreign bodies are interpreted by an experienced radiologist, they are highly suggestive of DF infective complications [10].

### 3.2. Magnetic Resonance Imaging

After initial radiography, MRI with fluid-sensitive, fat-suppressed sequences (i.e., short-tau inversion recovery [STIR] or fat-saturated T2-weighted images) is the modality of choice for investigating OM and associated soft-tissue complications [42,43] with high sensitivity and high specificity (90% and 83%, respectively) in the diagnosis of OM [38,44]. Post-contrast images improve the evaluation of soft tissue pathology, as they help in detecting abscesses and sinus tracts more easily [43]. Moreover, its radiation-free assessment becomes particularly important in the young population and when repeated follow-up imaging is likely to be necessary. However, standard MRI is typically based only on morphologic sequences, which provide only structural information. In the last years, technical improvements have allowed the capability to add functional quantitative information to structural information. The application of Dixon sequences improves image quality and increases the detection of sinus tracts and intraosseus sequestrums [45]. Diffusion-weighted imaging and the apparent diffusion coefficient value can help in the differentiation of diabetic neuropathic osteoarthropathy from OM with excellent inter-observer agreement [45].

**Figure 2:**
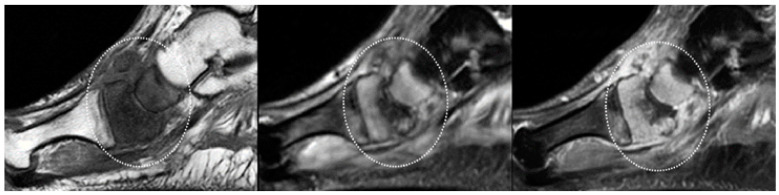
OM of the scaphoid and medial cuneiform. (From left to right) Sagittal T1-weighted, T2-fat-suppressed, and post-contrast T1-weighted fat-suppressed MRI clearly show that the marrow in the scaphoid and medial cuneiform has a low signal on a T1-weighted image (circle in left panel), increased signal on a fluid-sensitive fat-suppressed image (circle in middle panel), and post-gadolinium enhancement (circle in panel). These findings are indicative of OM.

Bone marrow (BM) with normal signal intensity excludes the diagnosis of OM in diabetic patients with STIs. Early OM is characterized by BM edema with low marrow signal intensity on T1-weighted images, high marrow signal intensity on fluid-sensitive fat-suppressed sequences, and post-contrast enhancement (Figure 2).

However, there are several mimickers of diabetes-related OM that may present problems to making a correct MRI diagnosis by showing BM edema and post-contrast enhancement. Furthermore, these conditions, including biomechanical stress changes related to altered weight bearing, recent post-operative surgery, inflammatory arthritis, and primarily neuropathic osteoarthropathy, may coexist with OM, further complicating the ability to make an accurate diagnosis. Consequently, if marrow edema is used as the primary diagnostic criterion, MRI may not be very specific.

Several secondary features such as subtending skin ulcer, sinus tract, abscess, tenosynovitis, or septic arthritis tend to be associated with OM. Their presence strongly suggests that osteomyelitis is present and can improve diagnostic accuracy [42,46].

**Figure 3:**
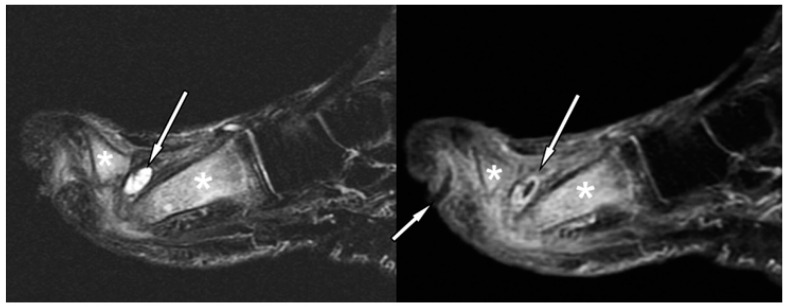
Forefoot ulcer, sinus track, and abscess associated with OM in a 57-year-old diabetic man with a 16-year history of insulin-dependent diabetes. Sagittal T2 fat-suppressed (left panel), and post-contrast T1-weighted fat-suppressed MRI (right panel) shows a dorsal thick rim-enhancing abscess adjacent to the first metatarsal head (arrows). Note a plantar ulcer appearing as a focal skin interruption and a sinus tract with rim-like enhancement (small arrow in right panel) extending near the first proximal interphalangeal joint. Given these findings, the hyperintensity (** in left panel), and post-contrast enhancement (** in right panel) in the first metatarsal, and proximal phalanx respectively, are indicative of OM.

- Skin ulcer: Skin ulceration is typified by focal interruption of the cutaneous line, with raised margins (secondary to preexisting callus formation). Acute ulcer appears hyperintense on fluid-sensitive fat-suppressed images, with marked peripheral post-contrast enhancement, which is a finding that is indicative of granulation tissue at the base of the ulcer. Chronic ulcer may be associated with fibrous healing and thus appears as a mass with low signal intensity on T1-weighted images and low to intermediate signal intensity on fluid-sensitive fat-suppressed images [42,43,46].
- Sinus tract and abscess: Sinus tracts and abscesses are some of the major findings in osteomyelitis. Morrison et al. determined the usefulness of primary and secondary MRI signs of OM and found that the identification of a sinus tract showed high specificity (average, 85%) for the diagnosis of osteomyelitis in the adjacent bone [47]. Sinus tracts typically extend from skin ulcers to tendon sheaths, bones, or joints, and they represent a route for the subsequent spread of infection leading to abscesses, septic tenosynovitis, and/or osteomyelitis [47]. Sinus tracts appear as linear fluid signal intensity on fluid-sensitive fat-suppressed images and display a characteristic “tram-track” pattern of the enhancement on contrast-enhanced images. The latter are the most sensitive MRI feature for detecting sinus tracts (Figure 3). Abscess is seen as a focal fluid collection that is hypointense on T1-weighted images and hyperintense on fluid-sensitive fat-suppressed images, with a thick rim post-contrast enhancement, due to the presence of granulation tissue (Figure 3). The presence of rim enhancement is essential in distinguishing abscesses from cellulitis or phlegmons, which present diffuse post-contrast enhancement [42,43,46].
- Septic tenosynovitis: Septic tenosynovitis generally results from the contiguous spread of infection from an adjacent ulcer, abscess, or sinus tract. On MRI, it is characterized by an abnormal increase in fluid within the tendon sheath, and post-contrast images may show a thick rim enhancement around the tendon, due to inflamed synovium. The tendon loses its constant low signal intensity and becomes thickened and indistinct [42,43,46].
- Septic arthritis: Similar to OM and tenosynovitis, septic arthritis occurs also as a result of contiguous spread from an adjacent ulcer, abscess, or sinus tract. No single MRI feature can differentiate septic from nonseptic arthritis; increased joint fluid and synovial thickening with contrast enhancement may also be seen in non-infectious inflammatory arthropathies. However, in pedal infections, the diagnosis of septic arthritis may be more specific if an ulcer and adjacent soft-tissue infection directly abut the joint, or, a sinus tract extends into the joint. Septic arthritis may demonstrate edema with post-contrast enhancement in adjacent soft tissue and on both sides of the joint. Reactive BM oedema, secondary to septic arthritis, should be differentiated from a superimposed OM. A low signal intensity on T1-weighted images, and proximal extension of subchondral edema beyond the subchondral bone usually indicate OM [42,48].

Distinguishing OM from neuropathic osteoarthropathy, in the absence of secondary signs of infection, is a common and difficult clinical and radiological problem. An accurate differentiation is mandatory, because the early detection of OM is essential to initiate prompt medical and/or surgical treatment. The location and distribution of anatomical changes may be helpful. Indeed, neuropathic osteoarthropathy usually involves the tarsometatarsal and metatarsophalangeal joints, while OM mostly involves the calcaneum, malleoli, and forefoot [49]. The biggest diagnostic problem arises in the midfoot. In this region, MRI findings may be inconclusive, and secondary signs of infection are invaluable in determining the presence of OM. Furthermore, neuropathic osteoarthropathy is primarily an articular disease; thus, BM oedema is limited to juxta-articular locations, whereas OM, which almost invariably results from an ulcer or abscess in contiguous soft tissue, shows diffuse marrow changes (Figure 3) [38,50].

The differentiation of infected from non-infected neuropathic osteoarthropathy remains extremely challenging, as the clinical and radiological findings may overlap. However, several MRI findings may be useful for distinguishing between these two conditions. Sinus tract formation, the replacement of soft tissue fat, fluid collections and diffuse marrow abnormality, diffuse joint fluid enhancement, and joint erosion support superimposed infection [43,51]. Thin rim enhancement of effusion, the presence of subchondral cysts, or intraarticular bodies indicate the absence of infection [19]. Bones that “disappear” on T1-weighted images and then “reappear” on contrast-enhanced or T2-weighted images (the “ghost sign”) is another MRI feature that indicates the presence of a superimposed infection. In uncomplicated neuropathic osteoarthropathy, the “ghost sign” is absent because there is bone destruction, but there is no infiltration of the marrow by inflammatory cells resulting in absence of the “ghost sign” [42,43,46].

## 4. Nuclear Medicine Imaging for DFI

NM techniques offer the possibility to image a process from a functional point of view, and they allow the identification of pathophysiological changes even before they become clinically detectable. Several radiopharmaceuticals are available for imaging infection and inflammation for both single photon emission computed tomography (SPECT) and positron emission tomography (PET) modalities, and most of them are currently applied for the diagnosis and follow-up of DFI.

### 4.1. Gamma-Camera Imaging for DFI

**Figure 4:**
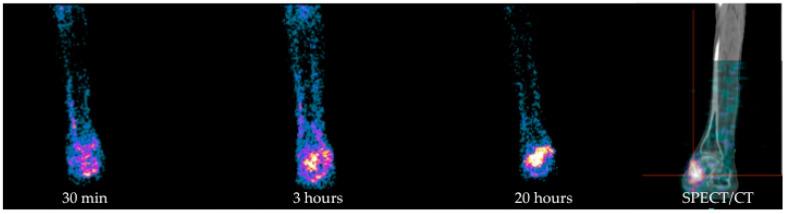
Example of ^99m^Tc-HMPAO WBC scintigraphy in a patient with a skin ulcer in the medial right malleolus region, previous amputations of left leg, and of all metatarsal heads of the right foot. From left to right: Planar images acquired after 30 min, 3 h, and 20 h p.i. of radiolabelled autologous leukocytes show an increased amount of activity over time in terms of intensity and extent, being consistent with OM with an involvement of adjacent soft tissues. Further SPECT/CT acquisition (right panel) correctly localized the uptake in the right talus and accurately evaluated its extent into surrounding soft tissues. HMPAO: hexamethylpropylene amine oxine, WBC: white blood cell.

Radiolabelled white blood cells (WBC) scintigraphy using both ^111^In or ^99m^Tc represent the NM cornerstone for the diagnosis of infection, since it specifically targets activated granulocytes, thus representing a surrogate marker of bacterial infections [52]. Several guidelines have been published by the European Society of Nuclear Medicine (EANM) with the aim to standardize labeling procedures, acquisition protocols, and interpretation criteria in all the centers [53,54,55]. In particular, to provide an in vivo imaging of the physiologic dynamic process of migration of granulocytes into the infective site, it is recommended to acquire images with times corrected for isotope decay at three time points after the reinjection of autologous cells. Once correctly acquired and displayed, the correct interpretation derives from the comparison of uptake extent and intensity between late images, acquired 20 h (h) post injection (p.i.) and delayed images (3 h p.i.). By following these recommendations, we can easily differentiate between a bone infection from a sterile inflammation. Indeed, in the first situation, the uptake increases over time in terms of extent and/or intensity, whereas in inflammation, the uptake decreases or remains stable over time [55,56,57]. By using these recommendations, and when combined with SPECT/CT acquisitions for the evaluation of the extent of the process and for the precise localization of the uptake, this modality reaches a very high accuracy in diagnosing an infection [58] (Figure 4). In a recently published meta-analysis and systematic review comparing the diagnostic performance of WBC scan, Fluorine-18 Fluorodeoxyglucose positron emission tomography ([^18^F]FDG PET/CT) and MRI for the detection of DF osteomyelitis (DFO), the pooled sensitivity and specificity of radiolabelled WBC were respectively 91% and 92% for ^99m^Tc hexamethylpropylene amine oxine (HMPAO) and 92% and 75% for ^111^In-oxine. In particular, ^99m^Tc-HMPAO WBC scintigraphy, followed by [^18^F]FDG PET/CT, showed higher specificity than other imaging modalities in the diagnosis of DFO, whereas the sensitivities were similar (approximately 90% for all) [59].

However, data regarding the use of radiolabelled WBC scintigraphy in DF are very discordant in the literature [60]. The sensitivity and specificity of this modality range from 75% [61] to 100% [62,63,64] and from 67% [64] to 100% [65] respectively, depending on deviation from the suggested labeling procedure, interpretation criteria adopted and, of course, different acquisitions protocols. In particular, several papers adopted only one-time point images, while others adopted outdated protocols of acquisition using fixed times or a fixed count, thus reflecting a wide heterogeneity of approach and results [64,66,67,68,69]. Hybrid imaging with SPECT/CT has also a great role in determining the accuracy of radiolabelled WBC scintigraphy, especially in discriminating superficial STIs from deeper infections. This differentiation is not easy achievable by using only planar images, but it is crucial for the correct management of the patient. Indeed, the primary goal for a correct therapeutic intervention derives from an accurate diagnosis of foot complications and, in particular, from the differentiation between sterile inflammation, STI, OM, and Charcot foot with or without a superimposed infection.

In this optic, radiolabelled WBC scintigraphy is the most accurate NM imaging modality able to achieve this differential diagnosis, since it provides an in vivo demonstration of the pathophysiology that underlies inflammatory and infective processes. However, the accuracy of radiolabelled WBC in differentiating OM from STI also depends on the district of the foot [60]. Despite previous considerations may be applied for a correct discrimination between these two conditions in forefoot disorders, in mid- and hindfoot, the presence of Charcot osteoarthropaty may also be considered. In this situation, radiolabelled WBC uptake could also be related to physiological BM expansion secondary to chronic inflammation, thus resulting in a lower specificity of this modality [70,71,72]. Therefore, in order to overcome this limitation and to improve the accuracy of WBC scintigraphy, it is suggested to perform an additional bone marrow scintigraphy (BMS) using nanocolloids. Indeed, both radiopharmaceuticals accumulate in BM but only WBC accumulate in infective foci, so if the images of these two modalities are congruent (match), the diagnosis of Charcot is the most probable; conversely, in case of mismatch (positive at WBC scintigraphy and negative at colloids), the diagnosis of OM may be done.

Despite radiolabelled WBC scintigraphy still representing the NM gold standard for the diagnosis of infections, some practical and technical issues, unfortunately, limit its use in all the centers. Indeed, this modality requires qualified personnel, adequate laboratories, and equipment. Moreover, it is a time-consuming procedure for both labeling and images acquisition, since acquisition at three time points is necessary. However, its accuracy has no peers in this field, and the availability of closed and single use kits has simplified the separation and labeling procedures, making all the steps safer for the operator [73].

The use of monoclonal antibodies (MoAbs) or antibodies fragments (Fab’) direct against specific antigens expressed by activated granulocytes has been proposed as an alternative to radiolabelled WBC scintigraphy, but they also have several cons mainly related to the high molecular weight of the entire antibodies that constitutes a limiting factor for their diffusion into the infective focus, their long plasma half-life, and their non-specific accumulation into inflamed sites. Furthermore, MoAbs induce human murine antibodies (HAMA) in the host, thus limiting their use at only one time in the life. Moreover, the role of MoAbs or Fab’ fragments has not been extensively investigated in DF, and data in the literature are mainly based on small groups of patients [74,75,76]. Moreover, at the moment, there are no standardized protocols for the acquisition and interpretation, and the few data in literature are not sufficient to conclude that MoAbs or their fragments have to be preferred to radiolabelled WBC scintigraphy in the assessment of DF disorders.

### 4.2. PET/CT Imaging for DFI

In the last decades, [^18^F]FDG PET/CT has gained an important role also for several indications in the field of infection and inflammation as specifically summarized in the guidelines published in 2013 by EANM and Society of Nuclear Medicine and molecular Imaging (SNMMI) [77].

[^18^F]FDG offers several advantages over conventional scintigraphy. First of all, it avoids the manipulation of potentially infected blood; secondly, the acquisition time is considerably shorter than radiolabelled WBC, and thirdly, the images’ quality resolution is better than those obtained with planar scintigraphy. Moreover, in the presence of CT co-registration, it is possible to have a precise definition of the anatomical landmarks, therefore evaluating the extent of the infective process into soft tissues or bone. However, [^18^F]FDG accumulates in infections, inflammations, malignancies, reparative processes, and in all the other conditions in which the glucose is metabolized as a source of energy.

In a meta-analysis published in 2013, the per-patients-based analysis showed a pooled sensitivity of 74% and a specificity of 91% [78]. Nevertheless, this meta-analysis was conducted only on 4 studies. Another more recent meta-analysis including 6 studies on 254 patients, in which the sensitivity and specificity of [^18^F]FDG PET/CT were 89% and 92%, respectively [59]. CT co-registration, of course, has a great influence on the accuracy of this imaging modality, but it also relies on correct interpretation criteria for a [^18^F]FDG PET/CT scan that, unfortunately, are not still well defined and standardized.

In a large cohort of 110 diabetic patients with suspected pedal OM, Nawaz et al. compared [^18^F]FDG PET and MRI. In this series, the first modality was less sensitive (81% versus 91%) but more specific (93% versus 78%) and accurate (90% versus 81%) than the second [79]. In this study, the diagnosis of OM was based on visual assessment of [^18^F]FDG uptake on bony structures without any semi-quantitative analysis of maximum Standardized Uptake Value (SUVmax). Furthermore, no CT co-registration was performed in this study, which may be influencing the relative low sensitivity compared to MRI.

Basu et al. explored the role of semi-quantitative analysis with SUVmax on 63 patients with DF disorders [80]. Patients with OM showed higher SUVmax values than patients with Charcot and uncomplicated DF, thus concluding that SUVmax could be a good parameter for differentiating these conditions. Although these findings were confirmed by other groups, some others did not find any correlation between SUVmax values and the different DF complications [81].

**Figure 5:**
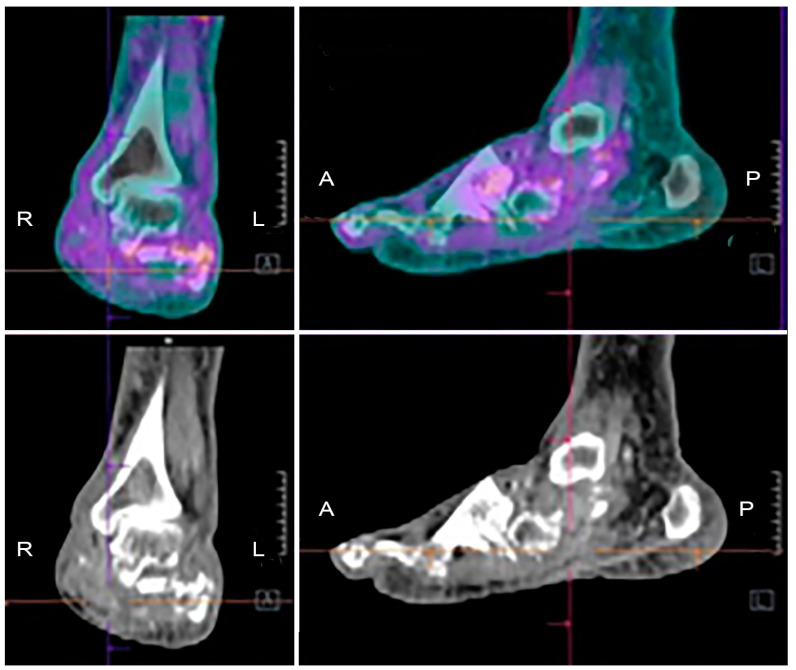
Example of [^18^F]FDG PET/CT in a patient with Charcot osteoarthropathy. Fused images (upper panels) show moderate and diffuse uptake, which is interesting in particular bones and joints of the mid and hind-foot. Co-registered low-dose CT images (lower panels) show the evident destruction of bony architecture. These findings are consistent with the diagnosis of Charcot foot, but they do not allow discriminating a pan-inflammation from a possible superimposed infection.

So, concluding, at present, well-defined interpretation criteria for differentiating infection, inflammation, STI, OM, and Charcot do not exist yet for [^18^F]FDG, thus representing a great limiting factor for this specific clinical indication. CT co-registration, although useful for localizing the uptake into bone rather than in soft tissue, does not solve the problem of discriminating an infection from inflammation/degeneration [82] (Figure 5).

Aiming to develop a more specific radiopharmaceutical for PET imaging, WBC have also been labeled with [^18^F]FDG, but published studies on DF still do not exist in literature.

## 5. Consensus Statements Emerged from Round Table of 3rd European Congress of Infection and Inflammation

During the 3rd European Congress of Infection and Inflammation organized in Rome in December 2019, several specialists evaluating patients with DF complications gave their lectures on this topic from different points of view. Here, we summarize several statements that emerged from the following round table, aiming to provide evidence-based answers to the most frequent clinical questions.

### 5.1. Is Radiography Useful in a Patient with Suspected OM?

Radiography should be the first-line imaging modality when evaluating for bone involvement in the DF. This approach is cheap, widely available, and associated with minimal harm. It provides an anatomic overview of the area of interest and any preexisting conditions that could influence the selection and interpretation of subsequent imaging modalities. Although we are not aware of any studies of the role of serial radiographs to diagnose OM, useful information can be obtained by performing serial radiographs to detect progressive bony changes.

### 5.2. Is a Negative Radiographic Examination Enough to Rule Out OM?

From a radiological point of view, a negative radiographic examination is not enough to rule out OM, since it is not sensitive in the detection of early stages of acute OM [10]. Radiographs may remain unremarkable for up to four weeks after the onset of infection. Furthermore, when radiographic changes of OM such as demineralization, bone resorption, and periosteal reaction become detectable, they may be difficult to be correctly interpreted because similar abnormalities may occur with Charcot osteoarthropathy and other disorders such as gout [40]. Therefore, the appeal to advanced imaging is madatory in order to achieve an accurate diagnosis.

### 5.3. Is MRI Indicated Since the First Diagnostic Steps?

MRI is not appropriate as first line imaging modality to diagnose OM; however, it is strongly recommended as an additional modality after initial radiography, when OM is suspected. MRI provides excellent spatial resolution and precise anatomical details; it allows preoperative mapping of the extent of infection, thus being helpful in minimizing the area of resection. Moreover, its radiation-free assessment becomes particularly important in the young population and when repeated follow-up imaging is necessary, and it is now widely available and less expensive than other imaging modalities [38].

### 5.4. Is MRI Indicated for Therapy Evaluation?

There is no relevant literature to support the use of MRI in the follow-up of DFO. However, this imaging modality can be very appropriate for determining whether the patients healed from the infection after treatment. Given that normal marrow signal reliably excludes OM [42], this condition should not be considered “cured” until there has been no evidence of recurrence for at least a year [83]. The radiation-free assessment as well as the high sensitivity and specificity for determining the presence or absence of pedal OM and STI [44] makes MRI imaging very suitable as a follow-up imaging modality, especially in young people.

### 5.5. Is WBC Scintigraphy Able to Differentiate between Superficial or Deep Infection?

The main disadvantage of planar NM imaging techniques is the limited spatial resolution and the lack of anatomic landmarks, which is especially a problem in the foot, where all the bony structures are very small and close each other. Indeed, an uptake on the soft tissues at planar images may overlap the underlying bone and vice versa, leading to a wrong interpretation of the scan and consequently to a wrong treatment. Therefore, as previously mentioned, the appeal to hybrid images is mandatory in order to improve the diagnostic accuracy of planar images.

Several authors explored the added value of SPECT/CT in the diagnosis [66,67,84,85,86] and therapy monitoring of DFO [87,88] and, despite the different protocols of acquisition adopted among the different studies, all authors concordantly agree that hybrid imaging is able to better localize the uptake into bone or soft tissues with an excellent definition of the extent of the infective process. Przybylski et al. reported a sensitivity, specificity, and diagnostic accuracy of ^99m^Tc WBC scintigraphy with SPECT/CT were 87.5%, 71.4%, and 80% respectively [85]. Heiba et al. examined 272 patients by using a combined approach with ^111^In WBC scintigraphy and bone scan [66], concluding that dual isotope SPECT/CT was superior than bone scan or WBC scintigraphy with SPECT/CT alone in discriminating STI from OM and, in another paper, they concluded that this combined approach is associated with a reduced length of hospitalization [67]. In the series studied in 2009 by Filippi et al., the interpretation of planar images substantially changed with the addition of SPECT/CT in 52.6% of cases, being able to rule out the infection in 6 cases, to diagnose OM in 1 case and to better define the extent of the process in 3 cases [86].

Therefore, concluding, data in the literature support the use of SPECT/CT in addition to planar images in the evaluation of DFI, in order to better localize the infection into bone or soft tissues and to accurately assess the extent of the process.

### 5.6. Can [^18^F]FDG PET/CT be Used as an Alternative to WBC?

The answer to this specific question is not easy, because it mainly depends on the local center’s equipment and facilities. As previously mentioned, the labeling procedures of leukocytes requires classified environments and laboratories and with isolators or class A wood with laminar flow, depending on local regulations. Personnel must be specifically trained to perform this procedure and must attend certified courses, thus impacting the department’s costs. Moreover, it is a time-consuming procedure that requires multiple times-point acquisition, and therefore, the patients need to come back to the hospital the day after in order to complete the examination. Aiming to overcome these limitations, several authors suggested the use of alternative approaches and [^18^F]FDG PET/CT, of course, represents the most attractive one due to higher quality images, shorter length of execution, and the easier and quicker length of handling of radioactive compounds, which does not require the manipulation of potentially infected blood. However, the well-known low specificity of [^18^F]FDG in differentiating an infection from a sterile inflammation and the lack of unanimous consensus on interpretation criteria make the diagnosis uncertain in most cases.

One paper published in 2011 by Familiari et al. [81] perfectly fits this question. In a small cohort of 13 patients with suspected OM, they compared [^18^F]FDG PET/CT and planar images of ^99m^Tc-HMPAO WBC scintigraphy, acquiring both modalities at three times point and using qualitative and semi-quantitative criteria of interpretation with SUVmax and Target/Background ratio (T/B) ratio at each time point. They identified a cutoff of more than 2.0 at late images as the best interpretation criterion for WBC scintigraphy: an increase of this cutoff between 3 and 20 h was suggestive for OM, while a stable or decreased uptake over time was suggestive for STI. Similarly, for [^18^F]FDG PET/CT, the best criterion for defining an OM was a SUVmax greater than 2.0 at 1 and 2 h p.i. and increasing with time. Whereas, if the uptake remaines stable or decreased over time, the scan was suggestive for STI. By using these criteria, the sensitivity, specificity, and accuracy of radiolabelled WBC scintigraphy were higher compared with [^18^F]FDG PET/CT. Therefore, they concluded that radiolabelled WBC scintigraphy should not be replaced by [^18^F]FDG PET/CT.

In accordance with this view, we strongly recommend the use of radiolabelled WBC scintigraphy in suspected pedal OM, as it also emerged from a recently published retrospective multicenter study [89]. [^18^F]FDG PET/CT could represent a valid alternative if it is not possible to perform this imaging modality due to the limitations of the single center, but the interpretation of a PET scan, at the moment, really relies on personal experience, and therefore, the scans must be evaluated with caution.

### 5.7. Is SUVmax Evaluation Useful for the Correct Interpretation of by [^18^F]FDG PET/CT Scan?

PET provides better resolution images and allows easier quantification methods than SPECT imaging. With radiolabelled WBC imaging, it is only possible to calculate T/B ratios between delayed and late images and to assess whether there is an increase or decrease of this value. With SUVmax evaluation on PET imaging, it is possible to quantify the uptake of by [^18^F]FDG at the infectious focus. Therefore, several authors tried to assess whether the use of this SUVmax could be beenficial in order to better differentiate between sterile inflammation, STI, OM, and Charcot.

For example, Basu et al. found higher SUVmax values in patients with OM compared with patients with Charcot and uncomplicated DF (2.9–6.2 versus 0.7–2.4 versus 0.2–0.7), concluding that SUVmax could be a useful parameter for differentiating these conditions [80]. Kagna at al. found a statistically significant difference in SUVmax between OM and STI (6.7 ± 3.7 versus 4.4 ± 2.4) [90]. However, these results were not confirmed by other studies [79,81], which may be because several techinical and practical factors may influence the variation of SUVmax calculations among different centers. Moreover, universally recognised cutoff values to differentiate among Charcot’s neuropathy, OM, and STI have not been defined yet.

Therefore, as it also stands for oncologic diseases, there is currently insufficient evidence to recommend that SUVmax could be a reliable tool for discriminating among different foot complications.

### 5.8. Is It Possible to Perform Radiolabelled WBC Scintigraphy during an Antibiotic Treatment?

The issue that ongoing antibiotic treatment could influence the sensitivity of radiolabelled WBC scintigraphy is still a matter of debate, and the opinions are very contrasting. From some papers, it emerges that the diagnostic accuracy of radiolabelled WBC is not significantly affected by the administration of antibiotics [87,88,91,92]. In 2013, Glaudemans et al. retrospectively studied a large population of patients with prosthetic joint infection, and they did not find significant differences in terms of diagnostic performance between patients under antibiotic treatment and patients that were not receiving therapy. Although this study was not focused on DFI, it was in support of the idea that this imaging modality retains a high sensitivity and specificity in detecting residual disease, independently by the administration of antibiotics. [93]. Indeed, as also indicated in recently published EANM guidelines [55], “patients receiving antibiotic treatment should not be excluded a priori since reports regarding their effect on WBC scintigraphy give various results”.

However, not all NM physicians place very much trust in performing this exam during antibiotic treatment, because possible false negative scans may be observed. Therefore, despite a perfect timing to perform WBCs scintigraphy following antimicrobial therapy not being clearly indicated in the literature, it is often a common practice to delay the radiolabelled WBC scintigraphy until 2 weeks after therapy withdrawal or to repeat the scan, in case of doubts in patients receiving antibiotics 2 weeks later.

Data in the literature on therapy monitoring in DF are mainly based on small series and do not allow drawing definite conclusions, but preliminary results seem to encourage the use of radiolabelled WBC scintigraphy, especially with SPECT/CT acquisitions, for the assessment of treatment response [87,88]. Similarly, [^18^F]FDG PET/CT could be used to follow signs of inflammation in the foot that may be still present, although the patient is considered clinically recovered [94], but definitive evidences are still lacking in the literature.

### 5.9. Do We Need to Perform a Combined Bone Marrow Scintigraphy in Addition to Radiolabelled WBC Scintigraphy for the Evaluation of Charcot?

Charcot osteoarthropathy is a condition that further complicates the challenging diagnosis of DFI. Radiolabelled WBC uptake in mid/hind-foot must be always interpreted with caution considering the possible physiologic accumulation into expanded BM that is typically present in a Charcot foot, independently by the presence of an infection or not. Therefore, a BMS is strongly suggested in order to have a scintigraphic map of BM and to compare with WBC images. Palestro described two criteria to diagnose OM in the presence of Charcot’s arthropathy: (1) the presence of labeled leukocyte uptake without corresponding activity on marrow images and (2) the spatially incongruent distribution of two radiopharmaceuticals [70,71].

[^18^F]FDG also shows several limitations in the evaluation of Charcot, because the uptake in this condition is usually very intense and diffuse involving all the tarsal and metatarsal joints, reflecting the evident changes in bony architecture typical of this conditions. Therefore [^18^F], FDG is not able to discriminate whether Charcot is infected or not.

## 6. Conclusions

An accurate identification and differentiation among different types of DFI still represent a challenge for the clinician. The appeal to multimodality imaging and a multidisciplinary approach are mandatory in order to plan the most appropriate therapeutic strategy for the single patient. Several radiological and NM approaches are available, being MRI, radiolabelled WBC scintigraphy, and [^18^F]FDG PET/CT the most appropriate, but larger multicenter studies are still needed in order to create standardized diagnostic flow charts that could be applied worldwide.

## Figures and Tables

**Table 1:** Common interpretation criteria of different imaging modalities in diabetic foot infections.

	OM	STI	Charcot	Pitfalls
Radiography	Anatomical overview of the area of interest and any preexisting conditions that might influence the interpretation of subsequent procedures	Soft-tissue gas, and calcifications	Bony fragmentation, debris formation, subluxation/dislocation, bony fragments fusion, sclerosis of bone ends, fractures, osteophytosis, and deformity	Very poor sensitivity in the early stages of the diseases. BM edema cannot be detected
CT	Cortical erosions, periosteal reaction, small sequestra	Soft-tissue gas, and calcifications	No potential in acute condition. In chronic condition, CT may be acquired for a preoperative bone assessment	Very limited role in the imaging of DFI. BM oedema can not be detected
MRI	Diffuse BM involvement: decreased marrow signal intensity on T1-w images, increased marrow signal intensity on fluid-sensitive, fat-suppressed sequences, and post-contrast enhancement. Ghost sign	Identification of subtending skin ulcer, sinus tract, abscess, tenosynovitis	BM involvement is limited to periarticular locations	Poor discrimination between infection and sterile inflammation in Charcot
Radiolabelled WBC	Planar images: focal activity at 20–24 h that is often increased compared with the uptake at 3–4 h; SPECT/CT: uptake clearly associated with bone at CT	Planar images: focal/diffuse activity at 20–24 h that is often stable or decreased compared with the uptake at 3–4 h; SPECT/CT: uptake clearly associated with soft tissues at CT	Planar images: diffuse activity at 20–24 h that is stable or decreased compared with the uptake at 3–4 h; positive match with BMS; SPECT/CT: uptake clearly associated with bone destruction	Possible FN during antibiotic treatment or severe vascular disease
[^18^F]FDG PET/CT	Focal or diffuse uptake clearly associated with bone at CT	Focal or diffuse uptake clearly associated with soft tissues at CT without bone involvement	Diffuse uptake involving tarsal/metatarsal joints and bone destruction at CT	Poor discrimination between infection and sterile inflammation. FP in Charcot and. after foot surgery

BMS: bone marrow scintigraphy; CT: computer tomography; OM: osteomyelitis; STI: soft tissue infection; DFI: diabetic foot infection; WBC: white blood cells; BM: bone marrow; BMS: bone marrow scan; SPECT: single photon emission computed tomography; T1-w: T1-weighted; FN: false negative; FP: false positive.
